# Rapid Telehealth Implementation during the COVID-19 Global Pandemic: A Rapid Review

**DOI:** 10.3390/healthcare8040517

**Published:** 2020-11-29

**Authors:** Cristian Lieneck, Joseph Garvey, Courtney Collins, Danielle Graham, Corein Loving, Raven Pearson

**Affiliations:** 1 School of Health Administration, Texas State University, San Marcos, TX 78666, USA; 2 School of Health Sciences, Southern Illinois University-Carbondale, Carbondale, IL 62901, USA; joseph.garvey@siu.edu (J.G.); courtney.collins@siu.edu (C.C.); daniellegraham211@siu.edu (D.G.); coreinl@siu.edu (C.L.); ravenpearson@siu.edu (R.P.)

**Keywords:** telehealth, telemedicine, COVID-19, coronavirus, implementation

## Abstract

The implementation and continued expansion of telehealth services assists a variety of health care organizations in the delivery of care during the current COVID-19 global pandemic. However, limited research has been conducted on recent, rapid telehealth implementation and expansion initiatives regarding facilitators and barriers surrounding the provision of quality patient care. Our rapid review evaluated the literature specific to rapid telehealth implementation during the current COVID-19 pandemic from three research databases between January 2020 and May 2020 and reported using preferred reporting items for systematic reviews and meta-analyses (PRISMA). The results indicate the rapid implementation and enhanced use of telehealth during the COVID-19 pandemic in the United States surrounding the facilitators and barriers to the provision of patient care, which are categorized into three identified themes: (1) descriptive process-oriented implementations, (2) the interpretation and infusion of the CARES Act of 2020 telehealth exemptions related to the relaxation of patient privacy and security (HIPAA) protocols, and (3) the standard of care protocols and experiences addressing organizational liability and the standard of care. While the study limitation of sample size exists (*n* = 21), an identification of rapid telehealth implementation advancements and challenges during the current pandemic may assist health care organizations in the delivery of ongoing quality care during the COVID-19 pandemic.

## 1. Introduction

### 1.1. Rationale

Telehealth has existed in the health care industry since the early 1960s and continues to develop as emerging technologies advance in terms of their ability to care for patients. Telehealth can be as rudimentary as a provider-to-patient phone call regarding their current health status or take a more advanced approach, including synchronous webinars, the sharing of digital patient information, and associated administrative tasks. Since 2007, the World Health Organization (WHO) has defined telehealth as [1]:

“The delivery of health care services, where distance is a critical factor, by all health care professionals using information and communication technologies for the exchange of valid information for diagnosis, treatment and prevention of disease and injuries, research and evaluation, and for the continuing education of health care providers, all in the interests of advancing the health of individuals and their communities.”

While telehealth is a much broader term used in the industry that includes both clinical and non-clinical services provided remotely, the term telemedicine refers specifically to the provision of remote clinical (non-administrative) services [2,3]. Both telehealth and telemedicine have been determined to provide enhanced access to health care services, especially during the COVID-19 global pandemic and the need for physical distancing. Such practices and protocols vary between and even within health care organizations. The broader term telehealth encompasses the practice of telemedicine and therefore is referred to synonymously as “telehealth” for the purposes of this study and the related search criteria [2].

Telehealth technology continues to increase in usage across all health care industry segments and is becoming more accepted by many organizations and patients alike. The use of telehealth technologies to support health care organization reach, accumulation, and the communication of health care information has recently been enhanced by the COVID-19 global pandemic [4]. An increased effort to infuse or further enhance such technologies into daily organizational care processes and practices to maintain daily health care operations continues to occur, increasing routine provider visits in the USA by over 150% [4]. Examples of such technological applications already utilized by health care organizations include mobile phone apps, remote patient monitoring (RPM) equipment, and even health education services [2]. However, the researchers hypothesize that the initiative for health care organizations to implement or expand upon these and other telehealth applications during the pandemic present unique challenges and opportunities for all health care stakeholders.

### 1.2. Telehealth and COVID-19

With the rush to expand or even initiate telehealth services, the use of this technology continues to be adapted to decrease the number of patients who do not have the proper access to care while also supporting pandemic physical distancing requirements. Exacerbated by the ongoing updates regarding vulnerable populations, those seeking medical care from their provider and medical team members during a global pandemic can be supported by telehealth. This technology is often deemed the solution for a wide range of routine and other acuity levels of care. Recognized as a solution to the challenges presented by the pandemic, alterations to previous telehealth policies include a relaxing of HIPAA standards, increased waivers from the Center for Medicare and Medicaid Services (CMS) to expand eligible services, and even billing/reimbursement clarifications to support the utilization of telehealth services (Wavier-1135) [5].

The disruption in health care practice caused by COVID-19 continues to raise the demand for telehealth, and its use in the industry is projected to increase by 64.3% by the end of 2020 [6]. Due to the rapid adaptation and implementation of telehealth in the United States, the market is projected to display a staggering seven-fold increase in growth by the year 2025; this relates to a five-year compound annual growth rate of 38.2% [6].

### 1.3. Objectives

The purpose of this rapid review is to identify the main underlying constructs (themes) in telehealth implementation and utilization during the COVID-19 pandemic (January 2020 through to May 2020) to assist with ongoing and early-adopter experiences regarding telehealth implementation. The rationale behind this rapid review is to provide a framework for future research as ongoing telehealth initiatives continue to be implemented and adapted to the United States health care environment. This review and the identified underlying themes should help the health care industry’s ongoing goals to provide quality care, while also addressing rapid implementation facilitators and the barriers identified during the early stages of the COVID-19 pandemic.

## 2. Methods

### 2.1. Overview

The research process began with a review of the research team’s intent and associated health care terms and research protocols per the PRISMA review standard. Three main research databases were utilized (the Cumulative Index to Nursing and Allied Health Literature, PubMed/MEDLINE, and ScienceDirect). The PubMed/MEDLINE database was utilized in this study because “telehealth” is a recognized vocabulary term in the MeSH (Medical Subject Headings) controlled vocabulary thesaurus, which is utilized for indexing articles for PubMed and clearly identified in the research. CINAHL is a health database that indexes nursing and allied health literature from a range of topics highly applicable to the research topic. The ScienceDirect database was also included based on its contribution to the initial search criteria sample size via its Novel Coronavirus Information Center, providing free access to timely health and medical research publications that align with the research question.

### 2.2. Inclusion Criteria

Study search terms and Boolean operators generated the database search string that resulted in the identification of current telehealth research in response to the COVID-19 pandemic. The identification of key terms for the research databases continued with the use of “telehealth” while also adding “virtual care” to broaden this perspective and associated health care technology research articles. The sample was further qualified with the use of “Coronavirus”, “COVID-19”, and “2019-ncov” (as suggested by the EBSCO database search engine) search criteria keywords using Boolean search operators. The initial search results were 3128 articles published between 1 January 2020 and 26 May 2020. This date range was incorporated into the rapid review criteria to help ensure that only telehealth implementation and enhancement initiatives related to the COVID-19 global pandemic were included to support the research objective. The initial database query and identification of the article sample was conducted by the researchers in May 2020 and the results/analysis was conducted in June 2020.

### 2.3. Exclusion Criteria

Studies in this review were eligible if telehealth implementation was specifically addressed and reported in quality journals (peer-reviewed) and also met the January–May publication date range. Limited studies reported patient outcomes as a result of rapid telehealth implementation. Therefore, the research objective was to focus primarily upon the rapid implementation facilitators/barriers of telehealth during the pandemic. As long as telehealth implementation facilitators and/or barriers were addressed and other search criteria were met, the article was included in this rapid review. The decision to include a wide range of study methods in the sample was made by the authors to help improve the sample size of the rapid review, primarily due to the recent nature of the global pandemic and the relevant nature of the research topic.

**Figure 1:**
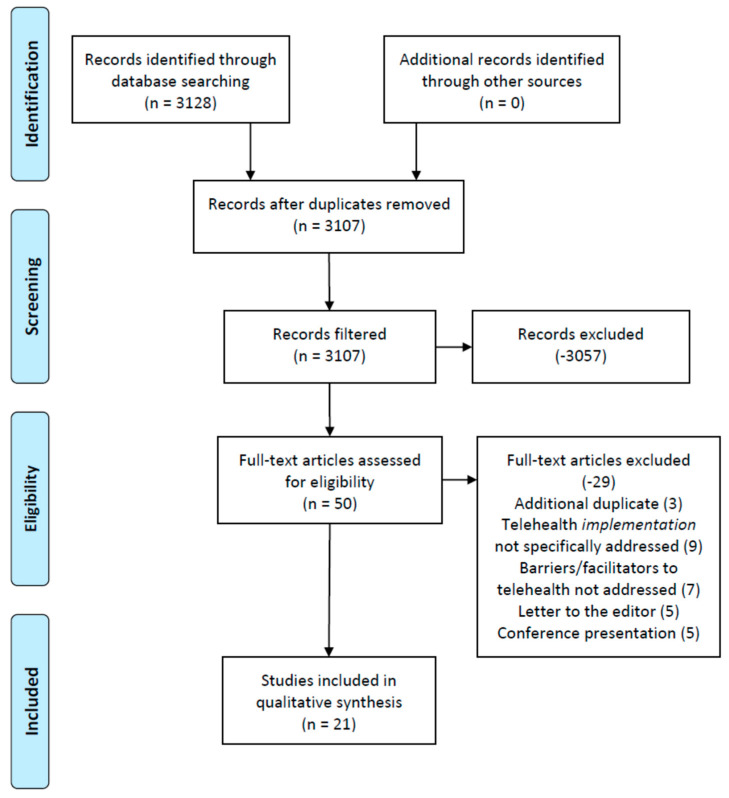
Preferred reporting items for rapid reviews and meta-analyses (PRISMA) figure that demonstrates the study selection process.

To produce focused, applicable results meeting the research objective, additional filters were applied to the research database findings, based upon each database’s online search platform filter options. For CINAHL, in addition to the 2020 publication date criterion, full-text, English only, and academic journals only criteria were applied, resulting in 29 articles. For PubMed, in addition to the publication date criterion, scholarly peer-reviewed journals only, linked full-text only, and English only criteria were applied, resulting in 16 articles. For ScienceDirect, in addition to the publication date criterion, research articles that were open access status only were queried and yielded 5 articles. Figure 1 illustrates the rapid review process and the applied search exclusion criteria.

A rigorous review of the 29 (CINAHL), 16 (PubMed), and 5 (ScienceDirect) articles was conducted by the authors by reading the full manuscripts of each article. This was accomplished by the six reviewers splitting into two separate groups to review the assigned articles to identify the underlying themes related to rapid telehealth implementation and expansion (Table 1). Articles were internally numbered; the first group (reviewers 1–3) read articles 1–30 and the second group (reviewers 4–6) read articles 11–50. Any article inclusion/exclusion discrepancies among a three-reviewer group finding were resolved by involving the other three reviewers’ assessment of the article; this instance occurred only twice during the review.

Overall, 19 articles were removed from CINAHL, 10 articles were removed from PubMed, and zero articles removed from ScienceDirect based on applicability to the research objective and germane to the overall study initiative. Specifically, the remaining items had to meet the research objective of the study and meet all the inclusion criteria by qualifying as peer-reviewed publications in academic journals and also directly addressing the implementation of telehealth during the COVID-19 pandemic. For example, while many articles met the exclusion criteria, during full-text screenings it was observed that several studies (9) were not targeted or primarily focused toward COVID-19 and the implementation or improvement of telehealth resources. Furthermore, seven (7) articles were also excluded from the study for not providing facilitators or barriers related to the implementation of telehealth during the pandemic (required data items to be observed by the research team during the article screening process). The rapid nature of this review regarding the rapid implementation of telehealth during the pandemic restricted the researchers’ efforts in data item observations to identify facilitators and barriers only in an effort to assist the medical community with the implementation and ongoing facilitation of telehealth efforts. Article selection bias was addressed through a series of consensus meetings (via webinar) that focused on each of the 10 article sets (Table 1) reviewed by the research team.

## 3. Results

Table 2 demonstrates the findings of the remaining articles (*n* = 21) with a description of the study participants (health care organizational type), the coding of the study design strength per the Johns Hopkins Nursing Evidence-Based Practice Model (JHNEPB) protocol listed at the end of the table, and a summary of the facilitators and/or barriers to rapid telehealth implementation identified by the researchers. Based upon these results, researchers were then able to identify and code all the underlying themes demonstrated in the sample. While (patient) outcomes were part of the researchers’ initial review process, this variable was found to not specifically be addressed beyond the implementation and facilitator/barrier findings with regard to telehealth. As a result, positive outcome findings were limited to the organizations’ abilities to conduct a rapid implementation and/or continuous improvement of telehealth services as an initiative to provide access to quality care during the pandemic.

The identified studies’ quality, as assessed through the use of the JHNEBP study design coding methodology, demonstrated that the majority of the literature (16 articles, 76% of the sample) falls within the level 4 category (opinion of nationally recognized experts based on research evidence and/or consensus panels). The remaining literature in the sample demonstrated a study design interpreted as level 3, non-experimental, qualitative, or meta-synthesis studies (4 articles) and 1 quasi-experimental (level 2) study design. While the strength of evidence regarding this review’s literature sample (primarily consisting of level 4 study designs) is important to note, the researchers came to the conclusion that this observation was possibly due to the nature and timeliness of this rapid review. A limited ability to conduct true experimental and/or randomized control trial (RCT) studies concerning the implementation of telehealth to date potentially exists. Finally, while a level 5 study (expert opinions not based on any research evidence) was identified earlier on in the researchers’ review process, this study was excluded because it was a letter to the editor or similar type of manuscript (Figure 1).

The health care organizations referenced in the literature were primarily located in the United States (19 of the 21 articles in the sample). Of the remaining two articles, only one specifically addressed telehealth implementation facilitators/barriers observed across multiple countries (USA, UK, and Australia) [7]. The researchers did not identify any telehealth facilitator or barrier variable outliers in their review of this literature, as compared to the rest of the review sample.

**Figure 2:**
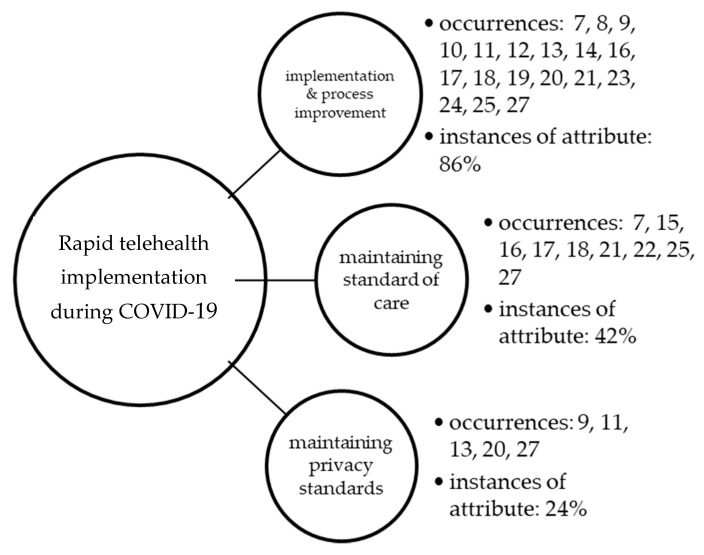
Occurrences of underlying themes as observed in the literature.

The rapid review process identified very recent, highly appliable telehealth publications that include the COVID-19 pandemic challenge to the health care system from a variety of health care organizations and the implementation and expansion of telehealth services. Of the total sample (*n* = 21), each researcher read assigned articles in the sample and all six reviewers worked to establish a consensus on the major themes (constructs) identified from their individual research initiatives. Each article was coded based on the theme(s) identified by the group, as shown in Figure 2. The percentage of occurrence of the overall construct is also shown, as compared to the total sample.

The most evident underlying construct in the rapid review was process improvement and descriptive facilitators/barriers identified as related to the implementation and continued use of telehealth during the COVID-19 pandemic (prevalent in 86% of all articles reviewed). Standard of care was the next common theme identified in the review, evident in 42% of articles in the sample. The maintenance of privacy standards was the third concept identified as most common in the review, with only five articles (24% of those reviewed) focusing on this important initiative. The identified themes (implementation and process improvement, maintaining the standard of care, and maintaining privacy standards) were not mutually exclusive, and therefore it was common for multiple constructs to be identified within any single article.

## 4. Discussion

### 4.1. Implementation and Process Improvement

The COVID-19 pandemic has led to many paradigm changes within the health care environment. One such change is the need to provide patient populations with high-quality health care services at a safe distance. The implementation of various virtual care modalities has been forced to take place at a rapid pace, causing many health care systems to either develop or restructure existing virtual care programs. Historically, health care programs have been slow to develop telehealth programs despite telehealth being known to reduce cost and increase patient access [28]. Programs began the implementation of telehealth by seeking out various virtual care services such as Zoom and Doximity and by researching existing electronic medical record virtual health systems [11,29]. In using these types of virtual visit applications, both patient and provider clinical processes needed to be reshaped and adjusted [17]. By adjusting their practices, health care systems were able to identify which telehealth strategies were both cost-effective and patient-friendly [17,28]. Applications which were identified and utilized by health care systems oftentimes were chosen due to their ease of use for various types of patient populations. Knowing that multiple different patient populations would be relying on telehealth, many of the simplest and easiest to use technologies were chosen as the best mediums for telehealth visits.

Similarly, most rapid implementations of virtual health have also involved the identification of appropriate computers, laptops, tablets, and smart phones for both patient and provider usage [17]. In the majority of circumstances, providers preferred the usage of desktop or laptop modalities for the visits, while patients preferred to use their home tablets or smartphones. Successes were found through the ease of the various applications and the devices being utilized for the appointments [17]. The potential success of rapid implementation of telehealth services during the COVID-19 pandemic may end up being attributed to the strengths of the applications being used by the health care systems.

The rapid implementation of telehealth services has also provided patient populations with a safe and effective treatment modality during the COVID-19 pandemic. Patient safety is a factor in the implementation of virtual visits. By having a virtual visit at a distance, patients feel that they are being cared for while in the safety of their own home [30]. While this service may not be a full alternative to an in-person visit, it does provide the patient with a feeling of convenience [28]. Virtual visits also are convenient for provider scheduling purposes. This convenience acted as a factor in patient and provider acceptance of usage of telehealth services. The implementation of telehealth services also provided virtual visit opportunities in the inpatient realm of health care services [17].

During the implementation of telehealth throughout COVID-19, health care systems encountered barriers to rapid implementation. Barriers such as low internet connectivity, lack of device access, elderly patient populations, and general technology difficulties slowed virtual visit implementation [25]. However, many of these barriers were able to be corrected by varying application usage, patient education practices, and provider/patient flexibility. Telehealth implementation also faced the barrier of lack of provider buy-in. Although older physician may face challenges with technology, they can be educated and trained to use devices and applications [29]. Competency with the use of technology and embracement of such contributions to the delivery of care continues to be an observed barrier in selected studies [7,9,10]. As the pandemic continues, many of these barriers should be addressed as health care systems gain more experience with telehealth.

### 4.2. Maintaining the Standard of Care

Since the onset of the COVID-19 pandemic, concerns surrounding the standard and liability of care formalities have heightened. Clinicians and policy makers have expressed inevitable controversy considering the legal liabilities associated with the current health care crisis [7,21,22]. When considering the scarce nature of health care resources and state activated crisis, liability protection becomes crucial. The rapid implementation of telehealth during the COVID-19 pandemic necessitates both timely and efficient regulations. As a result, both patient and provider experience are major factors when implementing telehealth policy changes [17,27]. There is also a prevailing focus to address the needs of individuals receiving care by way of telehealth, particularly centered around the principle of informed consent [17,18,21,25].

While the utilization of non-HIPAA compliant videoconference solutions has increased during the pandemic, significant concerns regarding patient privacy and private health information confidentiality remain a concern [28]. Mental health providers and supportive personnel (such as family members, etc.) stress limitations of telehealth communications to the extent that the overall patient status and other contributive observations are lacking or otherwise less noticeable [28]. This has caused a hesitance to take action due to concerns of liability that may resolve in death or injury. Many legal experts agree that the common law legal standard of care is adaptable to changing circumstances, and therefore would adjust to the necessities of medical care in a pandemic [28]. The standard care of liability has become increasingly controversial as USA health care systems continue to experience everchanging policy implementation at organizational levels.

### 4.3. Maintaining Privacy Standards

The adaptation of health care information technology to advance the use of telehealth capabilities comes with a growing need for proper privacy and security use and regulations. With the onset of the COVID-19 pandemic, delivering health care has come with new physical distancing and requirements, which telehealth is credited with enhancing [13,27]. Though telehealth is the safest and most reliable avenue to continue to treat patients, it does come with the concern of risking patient information, as well as protecting the patient’s privacy as outlined in HIPAA. As the HIPAA privacy rules have relaxed, the innovations connected to health technologies have increased, which has provided resolutions to the challenges in health care delivery [9,20,27].

Additionally, the research supports an interesting finding related to the other extreme: too much health privacy as a result of the implementation of telehealth [13,27]. This finding is further supported by an additional resource identified upon completion of the rapid review that discusses this phenomenon. Specifically, this study offers information regarding “…study participants [who] were more vulnerable to having potential anxiety and depression due to social isolation and misinformation about the virus” [30]. Additional information identifies the lack of social interaction in-person versus the telehealth communication methods as true to organic patient–provider interactions [30].

## 5. Study Limitations

This rapid review presents research limitations that are common in similar review studies. An intended limitation of this research was rating bias, which existed within individual researcher reviews of the sample articles. This limitation was addressed by group-level decisions and consensus was attained surrounding the thematic coding of each article. Research database findings will continue to change (therefore increasing the number of studies meeting the inclusion criteria) as research/publications continue to occur during the pandemic. The limited use of the database search criteria timeframe is also a study limitation. While sample size is an important limitation of the study, the focused research topic that identified initial themes as related to recent telehealth implementation and service expansion do support ongoing research surrounding identified constructs. As a result, the qualification and categorization of study design and related results as compared to a control group were limited in the available literature reviewed.

## 6. Conclusions

Changes are occurring in health care due to the COVID-19 global pandemic. Telehealth is continuing to adapt to environmental and industry needs in order to provide the highest quality of care for a variety of patients, while also assisting with physical distancing mandates and other public health measures. This research was initiated to rapidly identify underlying constructs in recently published literature related to the use and implementation and expansion of telehealth as applicable to the COVID-19 pandemic. Ongoing research surrounding the implementation of best practices, privacy concerns, and industry-specific (medical specialty) best practices is recommended to assist with the ongoing implementation and increased utilization of telehealth services. The patient experience of care and the specific health outcomes of care delivered via telehealth during the COVID-19 pandemic would also be opportune areas for future research. Health care organizations are encouraged to continue to infuse telehealth initiatives to support patient care and related outcomes during the COVID-19 pandemic with ongoing attention and efforts directed towards implementation/process improvement of best practices, controlling organizational liability by maintaining standards of care, and the maintenance of altered privacy standards during this challenging pandemic time period.

## Figures and Tables

**Table 1:** Reviewer assignment of the initial database search findings (full article review).

Article Assignment	Reviewer 1	Reviewer 2	Reviewer 3	Reviewer 4	Reviewer 5	Reviewer 6
Articles 1–10	X	X	X			
Articles 11–20	X	X	X	X	X	X
Articles 21–30	X	X	X	X	X	X
Articles 31–40				X	X	X
Articles 41–50				X	X	X

**Table 2:** Summary of findings (*n* = 21).

Author(s)	Participant(s)	* JHNEBP Study Design	Telehealth Facilitator(s)	Telehealth Barrier(s)
Fisk et al. [7]	Australia, UK, and USA governments and health care agencies	3	Patient choice, cost, and convenience cited. Reduction in hospital admissions. Improvement in patient health literacy.	Further requirement to become integrated within health and social care service frameworks. Not viewed by patients as a 100% suitable replacement for in-person health care treatment. Technology concerns/barriers/bandwidth.
Drogin [8]	Mental health providers	4	Cites decrease in travel costs, and shorter eval turnaround time occasioned by more flexible scheduling. Increased validity of FMTA evaluations due to virtual setting/administration of exam.	Consideration to circumstances in which face-to-face interactions are eliminated by pandemic situations. Questionable communication ‘behind the scenes’ with patient during online exams are of concern.
Green et al. [9]	Chiropractic providers within a physical medicine practice	4	Communicate the availability of chiropractic specialty (virtual) care to neighboring providers ahead of time. Modify encounter to enable patient to demonstrate posture/etc in front of camera.	To implement video visits effectively and legally, one must consider several requirements (privacy, legal, others). Reviews several potential problems associated with video visit delivery, yet also associated solutions to assist others in implementing video-based care.
Keihanian et al. [10]	Gastroenterology group practice	2	Provider preference for webinar over phone visits/alternatives for higher acuity or less technology-inclined patents/visits. Private practice (versus academic centers) more apt and willing to support rapid implementation.	Provider half-days increased, while number of total visits decreased (patient throughput). Technical issues and patient preparedness for virtual visits cited. Lack of medical training (residents/fellows) once changed to virtual visits (supervision concerns).
Carlson et al. [11]	Academic pediatric center	4	Availability to pre-announce future available appointments helps with scheduling logistics. Ability to create an online patient portal with clinician/parent information separation for clinical reasons. Ability for increased patient (child) confidentiality exists due to increased access (availability to log-on without parent if necessary).	Access remains a concern when requiring children to make themselves available online via webcam. Patient confidentiality and parent authorization concerns.
Imperatori et al. [12]	Mental health organization	3	Psychopathological symptoms that increased during the pandemic may be addressed via virtual reality (VR) applications at an enhanced level.	Significant/clear guidelines for the correct use of this technology within mental health practice. Several technological and access issues related to the adoption of VR.
Fang et al. [13]	Academic medical center	4	Minimized PPE usage in the virtual environment. Respiratory isolated patients were not as big of a concern in the virtual environment. Enabled all patients who have been affected by hospital visitor restrictions to connect with their families.	Device cost, privacy, and security cited as provider concerns. Free apps and other software offer limited and/or varying opportunities (piecemeal).
Wright et al. [14]	Neurosurgery group practice	4	Multiple potential advantages of continuing to expand this model of health care delivery in specific neuroscience modality treatments. Alternative settings for telehealth include the permitted use of Google Hangouts, Facebook Messenger video chat, and Apple FaceTime.	Preparations must also be made for the unintended consequences of increased neurosurgery availability (many are listed). Cost and administrative challenges are a significant barrier to multistate telehealth licensure. Increased time per visit is a concern.
Gould et al. [15]	Gerontology	4	Increased access to care, even when compared to before the pandemic. Helps/assists (but does not completely resolve) social isolation and loneliness.	Sensory impairment accompanying aging and older adults of concern. Online/virtual movements, body language not interpreted the same, plus generational differences. Device ownership comes with an assumed competency of the technology and this is not always the case.
Gao et al. [16]	Gerontology/physical activity/recreational activity specialty	4	Efficacy and effectiveness of VR exercise in the promotion of favorable health outcomes among the older adults VR-based interventions are most effective in promoting improved health outcomes among older adults. Weight-loss management programs demonstrated some effectiveness (required further study/investigation).	Access to gaming systems cannot be assumed. Enhanced sessions can help with motivation (immersed treadmill video, etc) and also help with isolation concerns.
Wosik et al. [17]	Variety of health care organizations, undisclosed.	3	Health care encounters can be custom-tuned with telehealth applications for a better experience and patient outcomes. COVID-19 phases (isolation, initial hospital surge, and post-surge) also call for different telehealth application.	Phases of the pandemic will affect how to respond with telehealth, and this can become tricky and problematic if not tuned accordingly. Privacy noted as a serious concern.
Jhaveri et al. [18]	Hospital-based psych/oncology service line.	4	Participation surged as the program became instantly accessible to more survivors. Use of common household items became popular in exercises/etc in conjunction with webcams.	Group discussion included heightened risk and fear of falling ill, health-related vigilance, and risk associated with potential delays in surveillance or other survivorship care. Concerns regarding the requirement to initiate the program for survivorship reasons (no choice) and speed of implementation. Minimal screen-sharing (privacy, etc).
Hewitt et al. [19]	Academic neuropsychology clinic.	4	Troubleshooting manual created out of mock webinar testing enabled effective implementation results. Validated protocols can be highlighted to diminish the interoperative neuropsychological assertion in an adversarial environment.	Testing modifications (non-standard) had to occur to administer inventories to remote patients. Questionable state-level reimbursement based upon a) payer type and b) patient location (in or out-of-state).
Chowdhury et al. [20]	Pediatric cardiology medical practice.	4	Virtual health helps to increase physical distancing requirements with children. Provides a good/better view into the patient’s home environment, versus only being seen in the clinic/in-person. Decreased wait times for access to care assist providers with in-person triage encounters.	Adapted staffing and billing models are required for better/future telehealth visits. Still does not substitute for in-person procedures required (EKG, etc). Vital sign medical equipment is not always available in the patient’s home, much less pediatric sizes.
Burgess et al. [21]	Mental health: bipolar disorder treatment protocols	4	Telehealth possible, increased access if patient is available. More concerns and best-practices to control liability, versus facilitators beyond physical distancing protocols.	Certain aspects of speech, affect, and psychomotor agitation may require more effort when delivering virtual care compared to in-person care. Telephone connections feature significant lag, it may be difficult to interpret apparent interruptions or changes in voice. It is important to not assume; instead, assess for changes in functioning given novel outlets for various habits in the time of COVID-19.
Fantz et al. [22]	Diabetes self-management.	4	Use of ongoing/continuous glucose monitoring with technology assists with limited PPE during the pandemic. Self-management promotes physical distancing. Ongoing use of these self-management devices allow for a much larger data collection.	Putting a sharp focus on self-management will require more patient responsibility. Costs related to continuous monitoring.
Gadzinski et al. [23]	Hospital urology patients (routine and emergency)	4	eConsults allow for better inter-provider communication and overall access to information. Conserves PPE and limits COVID-19 exposures. Allows for a centralized workforce that is inter-connected online to support patient care.	Telehealth visits can include focused physical examination maneuvers using image- and audio capturing devices to assess the dermatologic, cardiac, and pulmonary systems. Strong focus on telehealth waivers and liability concerns. eConsults with other medical providers come with additional billing/documentation requirements.
Shipchandler et al. [24]	Otolaryngology medical practice	4	Improve upon current telehealth systems Improvement on access to care. Avoidance of ‘high risk’ settings.	Each visit is scheduled for 20 to 30 min employees who work remotely from home. Challenges to continue remote care beyond pandemic (routine care).
Hirko et al. [25]	Large rural health system.	4	Continued third-party reimbursement is promising to help continue increased access to medical providers (often more than before the pandemic). Rural locations virus prevalence is not to be taken lightly as surges expected to increase.	Issues in broadband access in rural settings, which limit the reach and effectiveness of telehealth initiatives, must be prioritized. Often rural patients endure financial challenges limiting technology access.
Woo et al. [26]	Pediatrics obesity clinic.	4	Contact hours (online) for pediatric and family weight-management counseling and other programs help control the disparities experienced during the pandemic. Rapid weight-management lifestyle changes forced into virtual visits continue to allow for such contact sessions with patients/family.	Privacy concerns for patients enrolled in weight-management programs. Access and cost of webinar equipment is an issue. Technology will only assist and not alleviate food insecurities and health disparities for patients in weight management programs.
Moring et al. [27]	Mental health/PTSD provider organizations.	3	Cognitive processing therapy was not compromised/lowered throughout telehealth visits. Keeping track of patient locations (addresses) during various telehealth visits helps with unanticipated medical emergencies.	Practitioners should attempt to use platforms that provide secure, encrypted videoconferencing technology. There is an limitation to a provider’s ability to address non-verbal (or even verbal) cues to intoxication and other issues virtually, versus in-person visits.

* JHNEBP levels of strength of evidence (strength of study): Level 1, experimental study/randomized control trial (RCT); Level 2, quasi-experimental study; Level 3, non-experimental, qualitative, or meta-synthesis study; Level 4, opinion of nationally recognized experts based on research evidence/consensus panels; Level 5, opinions of industry experts not based on research evidence (not included in this study).

## References

[1] World Health Organization (2010). Telemedicine: Opportunities and Developments in Member States: Report on the Second Global Survey on eHealth.

[2] Mahoney M.F. (2020). Telehealth, Telemedicine, and Related Technologic Platforms: Current Practice and Response to the COVID-19 Pandemic. J. Wound Ostomy Cont. Nurs..

[3] American Academy of Family Physicians What’s the Difference between Telemedicine and Telehealth?. https://www.aafp.org/news/media-center/kits/telemedicine-and-telehealth.html.

[4] Centers for Disease Control and Prevention Emergence of the COVID-19 Pandemic—United States, January–March 2020. https://www.cdc.gov/mmwr/volumes/69/wr/mm6943a3.htm.

[5] Health and Human Services (2020). Telehealth: Delivering Care Safely during COVID-19.

[6] Imaging Technology News (2020). Telehealth to Experience Massive Growth Due to COVID-19.

[7] Fisk M., Livingstone A., Pit S. (2020). Telehealth in the Context of COVID-19: Changing Perspectives in Australia, the United Kingdom, and the United States. J. Med. Internet Res..

[8] Drogin E.Y. (2020). Forensic mental telehealth assessment (FMTA) in the context of COVID-19. Int. J. Law Psychiatry.

[9] Green B.N., Pence T.V., Kwan L., Rokicki-Parashar J. (2020). Rapid Deployment of Chiropractic Telehealth at 2 Worksite Health Centers in Response to the COVID-19 Pandemic: Observations from the Field. J. Manip. Physiol. Ther..

[10] Keihanian T., Sharma P., Goyal J., Sussman D.A., Girotra M. (2020). TeleHealth utilization in Gastroenterology (GI) clinics amid Coronavirus-19 (COVID-19) pandemic: Impact on clinical practice & GI training. Gastroenterology.

[11] Carlson J., Goldstein R. (2020). Using the Electronic Health Record to Conduct Adolescent Telehealth Visits in the Time of COVID-19. J. Adolesc. Health.

[12] Imperatori C., Dakanalis A., Farina B., Pallavicini F., Colmegna F., Mantovani F., Clerici M. (2020). Global Storm of Stress-Related Psychopathological Symptoms: A Brief Overview on the Usefulness of Virtual Reality in Facing the Mental Health Impact of COVID-19. Cyberpsychol. Behav. Soc. Netw..

[13] Fang J., Liu Y.T., Lee E.Y., Yadav K. (2020). Telehealth Solutions for In-hospital Communication with Patients under Isolation during COVID-19. West. J. Emerg. Med..

[14] Wright C.H., Wright J., Shammassian B. (2020). COVID-19: Launching neurosurgery into the era of telehealth in the United States. World Neurosurg..

[15] Gould C.E., Hantke N.C. (2020). Promoting Technology and Virtual Visits to Improve Older Adult Mental Health in the Face of COVID-19. Am. J. Geriatr. Psychiatry.

[16] Gao Z., Lee J.E., McDonough D.J., Albers C. (2020). Virtual Reality Exercise as a Coping Strategy for Health and Wellness Promotion in Older Adults during the COVID-19 Pandemic. J. Clin. Med..

[17] Wosik J., Fudim M., Cameron B., Gellad Z.F., Cho A., Phinney D., Curtis S., Roman M., Poon E.G., Ferranti J. (2020). Telehealth transformation: COVID-19 and the rise of virtual care. J. Am. Med. Inform. Assoc..

[18] Ma K.J., Cohen J.A., Ba M.B., Levin A.O., Goyal N., Bs T.L., Chesney M.A., Shumay D.M. (2020). “Soup cans, brooms, and Zoom”: Rapid conversion of a cancer survivorship program to telehealth during COVID-19. Psycho-Oncology.

[19] Hewitt K.C., Loring D.W. (2020). Emory university telehealth neuropsychology development and implementation in response to the COVID-19 pandemic. Clin. Neuropsychol..

[20] Chowdhury D., Hope K.D., Arthur L.C., Weinberger S.M., Ronai C., Johnson J.N., Snyder C.S. (2020). Telehealth for Pediatric Cardiology Practitioners in the Time of COVID-19. Pediatr. Cardiol..

[21] Burgess C., Miller C., Franz A., Abel E.A., Gyulai L., Osser D., Smith E.G., Connolly S.L., Krawczyk L., Bauer M. (2020). Practical lessons learned for assessing and treating bipolar disorder via telehealth modalities during the COVID-19 pandemic. Bipolar Disord..

[22] Fantz C.R., Rivers M. (2020). COVID-19 Awakens a New Focus on Surge Capacity Blood Glucose Testing and the Critical Role of Telehealth in Self-Management. J. Diabetes Sci. Technol..

[23] Gadzinski A.J., Andino J.J., Odisho A.Y., Watts K.L., Gore J.L., Ellimoottil C. (2020). Telemedicine and eConsults for Hospitalized Patients During COVID-19. Urology.

[24] Shipchandler T.Z., Nesemeier B.R., Parker N.P., Vernon D., Campiti V.J., Anthony B.P., Alwani M.M., Illing E.A., Ting J.Y. (2020). Telehealth Opportunities for the Otolaryngologist: A Silver Lining During the COVID-19 Pandemic. Otolaryngol. Neck Surg..

[25] Hirko K.A., Kerver J.M., Ford S., Szafranski C., Beckett J., Kitchen C., Wendling A.L. (2020). Telehealth in response to the COVID-19 pandemic: Implications for rural health disparities. J. Am. Med. Inform. Assoc..

[26] Baidal J.A.W., Chang J., Hulse E., Turetsky R., Parkinson K., Rausch J.C. (2020). Zooming Toward a Telehealth Solution for Vulnerable Children with Obesity During Coronavirus Disease 2019. Obesity.

[27] Moring J.C., Dondanville K.A., Fina B.A., Hassija C., Chard K., Monson C., Losavio S.T., Wells S.Y., Morland L.A., Kaysen D. (2020). Cognitive Processing Therapy for Posttraumatic Stress Disorder via Telehealth: Practical Considerations During the COVID-19 Pandemic. J. Trauma. Stress.

[28] Levine L.B., Guidry M. (2020). Telehealth and COVID-19. EP Magazine.

[29] Miller J.J. (2020). Jumping Into Telehealth. Psychiatric Times.

[30] Kojima N., Klausner J.D. (2020). Virtual House Calls: Telemedicine and Reforming the Health Care Delivery Model with Strategies Implemented in a Novel Coronavirus Pandemic. J. Gen. Intern. Med..

